# Scalable Nuclei Detection in HER2-SISH Whole Slide Images via Fine-Tuned Stardist with Expert-Annotated Regions of Interest

**DOI:** 10.3390/diagnostics15131584

**Published:** 2025-06-22

**Authors:** Zaka Ur Rehman, Mohammad Faizal Ahmad Fauzi, Wan Siti Halimatul Munirah Wan Ahmad, Fazly Salleh Abas, Phaik-Leng Cheah, Seow-Fan Chiew, Lai-Meng Looi

**Affiliations:** 1 Centre for Image and Vision Computing, CoE for Artificial Intelligence, Multimedia University, Persiaran Multimedia, Cyberjaya 63100, Selangor, Malaysia; zaka.mmu.my@gmail.com (Z.U.R.); wshmunirah@gmail.com (W.S.H.M.W.A.); fazly.salleh.abas@mmu.edu.my (F.S.A.); 2 Faculty of Artificial Intelligence and Engineering, Multimedia University, Persiaran Multimedia, Cyberjaya 63100, Selangor, Malaysia; 3 Institute for Research, Development and Innovation, IMU University, Bukit Jalil, Kuala Lumpur 57000, Malaysia; 4 Faculty of Engineering and Technology, Multimedia University, Bukit Beruang 75450, Melaka, Malaysia; 5 Department of Pathology, University Malaya-Medical Center, Kuala Lumpur 59100, Malaysia; cheahpl@um.edu.my (P.-L.C.); sfchiew@ummc.edu.my (S.-F.C.); looilm@um.edu.my (L.-M.L.)

**Keywords:** deep learning, digital pathology, human epidermal growth factor receptor 2 (HER2), silver-enhanced in situ hybridization (SISH)

## Abstract

**Background:** Breast cancer remains a critical health concern worldwide, with histopathological analysis of tissue biopsies serving as the clinical gold standard for diagnosis. Manual evaluation of histopathology images is time-intensive and requires specialized expertise, often resulting in variability in diagnostic outcomes. In silver in situ hybridization (SISH) images, accurate nuclei detection is essential for precise histo-scoring of HER2 gene expression, directly impacting treatment decisions. **Methods:** This study presents a scalable and automated deep learning framework for nuclei detection in HER2-SISH whole slide images (WSIs), utilizing a novel dataset of 100 expert-marked regions extracted from 20 WSIs collected at the University of Malaya Medical Center (UMMC). The proposed two-stage approach combines a pretrained Stardist model with image processing-based annotations, followed by fine tuning on our domain-specific dataset to improve generalization. **Results:** The fine-tuned model achieved substantial improvements over both the pretrained Stardist model and a conventional watershed segmentation baseline. Quantitatively, the proposed method attained an average F1-score of 98.1% for visual assessments and 97.4% for expert-marked nuclei, outperforming baseline methods across all metrics. Additionally, training and validation performance curves demonstrate stable model convergence over 100 epochs. **Conclusions:** These results highlight the robustness of our approach in handling the complex morphological characteristics of SISH-stained nuclei. Our framework supports pathologists by offering reliable, automated nuclei detection in HER2 scoring workflows, contributing to diagnostic consistency and efficiency in clinical pathology.

## 1. Introduction

Breast cancer is the most frequently diagnosed cancer and the second leading cause of cancer-related deaths among women globally [1,2]. Improving patient survival requires a multidisciplinary approach involving experts across pathology, medical, surgical, and radiation oncology [3]. Accurate diagnosis, staging, and grading of the disease depend on tissue biopsies, initially processed with haematoxylin and eosin (H&E) staining. For further insights, immunohistochemistry (IHC) staining is employed to detect specific protein receptors, such as the Human Epidermal Growth Factor Receptor 2 (HER2) [4]. HER2 overexpression, present in approximately 10% to 20% of breast cancer cases, is often associated with aggressive disease and a poorer prognosis [5]. However, HER2-positive patients benefit from targeted therapies, underscoring the need for accurate HER2 status evaluation [6,7]. When HER2 status is equivocal (score 2+), further assessment using in situ hybridization (ISH) techniques is necessary to determine HER2 gene amplification.

**Figure 1:**
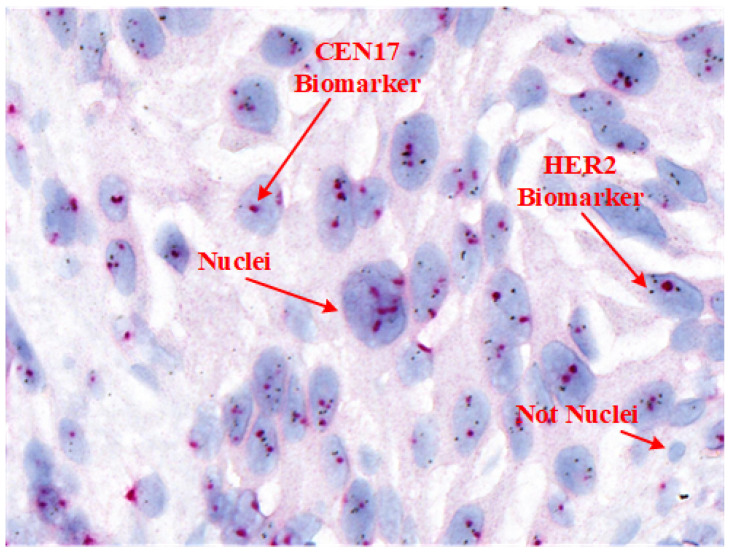
Anatomy of a histopathology HER2-SISH image, highlighting key features for HER2 and CEN17 signal analysis in nuclei segmentation.

While H&E and HER2 IHC provide essential insights, they do not confirm gene amplification. This assessment is conducted through ISH techniques, including fluorescence ISH (FISH), chromogenic ISH (CISH), and silver-enhanced ISH (SISH). HER2 protein overexpression is specifically evaluated by IHC [8]. In clinical settings, such as in Korea, IHC serves as the primary test for HER2, with SISH used as a confirmatory test for equivocal cases due to its accuracy, efficiency, and consistency compared to FISH and CISH. Table 1 provides a comparison of these techniques, and Figure 1 shows an example SISH image highlighting its key features.

Advances in digital pathology, such as whole-slide imaging (WSI) and cloud-based data storage, have transformed diagnostic workflows [9]. Integrating WSI data with telemedicine and big data analytics opens avenues for personalized healthcare and research-driven insights, incorporating data from epidemiological studies and genomic sequencing [10]. Computer vision techniques are crucial in digital pathology, as they enable automated and standardized image analysis, reducing observer variability and enhancing diagnostic efficiency [11,12]. As a fully automated staining method, SISH enables pathologists to analyze tissue morphology under bright-field microscopy, which is vital for accurate interpretation [13].

For accurate HER2 quantification in SISH, pathologists assess the ratio of HER2 signals (black spots) to centromere 17 (CEN17) signals (red dots) within selected areas containing 20 nuclei, following ASCO/CAP guidelines. However, manual nuclei segmentation in SISH WSIs is labor-intensive, as it involves variable nuclei shapes and distribution patterns essential for tissue and cancer characterization [14].

Automated nuclei segmentation poses challenges due to overlapping nuclei, staining variability, and cytoplasmic morphology differences. Traditional methods, such as intensity thresholding, marker-based watershed segmentation, and active contour models, are limited by computational demands or lack robustness [15,16,17]. Recent advances in machine learning, especially convolutional neural networks (CNNs), have demonstrated promise by learning features relevant to nuclei morphology and staining patterns [16,18].

Despite advancements, deep learning-based segmentation methods face limitations due to the lack of large, annotated datasets for training. To overcome these challenges, we propose a two-stage algorithm that combines a pretrained Stardist model with morphological preprocessing and fine-tuning on a novel, expert-annotated HER2-SISH dataset. This approach aims to improve nuclei detection accuracy in complex SISH images and streamline diagnostic workflows by offering automated tools for pathologists.

**Figure 2:**
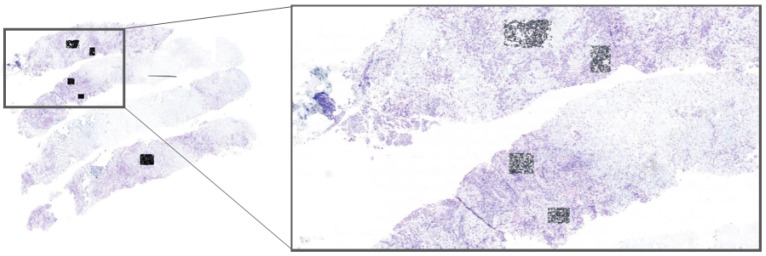
Visualization of selected regions of interest (ROIs) on a HER2-SISH whole-slide image (WSI) using the Cytomine platform. The black squares indicate expert-selected ROIs, where our fine-tuned Stardist model is applied for nuclei segmentation. This workflow enables pathologists to select ROIs on WSIs interactively, allowing real-time nuclei segmentation and accelerating the HER2 scoring process.

The primary contributions of this research include:

- Developing a two-stage framework that employs morphological preprocessing, a pretrained Stardist 2D Fluo model, and fine-tuning for precise nuclei detection in HER2-SISH histopathology images;
- Facilitating visualization and analysis of SISH-detected nuclei regions for enhanced clinical interpretation;
- Implementing our model within the Cytomine platform, allowing experts to select regions of interest (ROIs) directly from whole-slide images (WSIs) for real-time nuclei segmentation; this application, shown in Figure 2, enables pathologists to efficiently use our model for HER2 scoring, accelerating the evaluation process;
- Demonstrating the model’s effectiveness for automatic nuclei detection in complex SISH images, thereby supporting pathologists in HER2 assessment and scoring.

The paper is structured as follows: Section 2 reviews the literature on nuclei detection in histopathology. Section 3 describes our methodology, including datasets and training processes. Section 4 presents the experimental setup, and Section 5 provides a detailed analysis and discussion of results. Finally, the conclusion and future directions of this research are outlined in Section 7.

## 2. Related Work

In this section, we review the significance of nuclei regions in various histopathology-stained images, explore deep learning-based nuclei segmentation techniques for computational pathology, and discuss the role of artificial intelligence (AI) in addressing nuclei segmentation challenges. This review highlights the applications of deep learning and AI in digital pathology, especially as they pertain to automating complex and labor-intensive tasks in medical imaging.

### 2.1. Deep Learning-Based Nuclei Segmentation

Nuclei segmentation in histopathology images has been extensively studied, with numerous deep learning methods developed for this critical task. However, the inherent complexity of histopathology images continues to challenge the application of deep learning for nuclei segmentation.

Accurate nuclei segmentation is crucial for computer-assisted histopathology, as it underpins many downstream analyses essential for disease characterization and diagnosis. Yet, histopathology images present unique challenges: nuclei often form intricate structures such as clumps or nests, making precise annotation difficult. Furthermore, boundary delineation is complicated by the close proximity of epithelial cells and irregular mitotic patterns.

Addressing these challenges requires advanced deep learning techniques, innovative data augmentation strategies, and architectures capable of capturing the nuanced details of histopathology images. Collaborative data-sharing efforts within the research community are also essential to improve the performance and robustness of models for nuclei segmentation in histopathology. By overcoming these barriers, deep learning can enable more accurate and reliable computer-assisted pathology analyses, benefiting both clinicians and patients.

Several deep learning methods have been proposed for nuclei segmentation, often utilizing two-class probability maps followed by post-processing. For instance, ref. [18] introduced CNN-3C, which reframes nuclei segmentation as a three-class problem, achieving promising results across multiple organ types. Cui et al. proposed a fully convolutional network-based method for nuclei segmentation [19], while [20] formulated the task as a regression problem by predicting nuclei distance maps. Ref. [21] demonstrated that fusing networks led to better segmentation results compared to multi-task learning. Salvi et al. introduced a multi-scale adaptive nuclei segmentation (MANA) method, showing strong performance on multi-organ nuclei segmentation [22]. Recent work such as Xu et al. [23] explored transformer-based nuclei segmentation with improved generalization across staining variations. Similarly, Huang et al. [24] proposed a Multiscale CNN Transformer approach, showing strong results across nuclei segmentation. Despite these advancements, these methods often remain limited by the availability of sufficiently large, annotated datasets and the diversity needed to generalize across tissue types.

Adaptability to multiple organ types, clinical data sources, patient samples, and disease conditions requires large and varied datasets. Simple CNN architectures frequently encounter difficulties with overlapping nuclei, necessitating complex post-processing techniques. Many previous studies have approached nuclei segmentation as a classification or combined classification–regression task. We propose an alternative approach by treating it as a self-learning process that uses pretrained predictions to refine segmented nuclei regions. This method provides greater context awareness and global consistency by optimizing the loss function across the entire image rather than just at the pixel level, enhancing robustness and adaptability for various histopathology applications.

### 2.2. Stardist for Medical Imaging Applications

Accurate detection and segmentation of cell nuclei in histopathology images are critical for numerous medical applications, including cancer diagnosis and treatment planning. Factors such as low signal-to-noise ratios and clustered cells often complicate the identification of nuclei using traditional intensity-based algorithms.

To address these issues, Stardist [25,26] leverages deep learning techniques to enhance segmentation performance. Stardist applies to both 2D and 3D data, representing objects as star-convex polygons, which facilitates effective segmentation of individual cells in dense tissue sections [26].

A key innovation of Stardist is its use of a neural network to predict shape representations for nuclei in the form of star-convex polygons. This approach significantly improves segmentation accuracy. For each pixel within an object, the network predicts distances to the object boundary along multiple radial directions, effectively defining a star-convex polygon. This representation allows for accurate and reliable segmentation, even under challenging conditions, making Stardist a valuable tool for histopathology and other medical imaging tasks.

In addition to polygon prediction, Stardist incorporates object probability estimation to determine pixels belonging to nuclei, which contribute to shape estimation. To minimize redundant shape predictions, Stardist applies non-maximum suppression, eliminating overlapping shapes that likely represent the same object.

The approach outlined by [26] lies at the intersection of object detection and instance segmentation. While Stardist may not achieve pixel-perfect precision, it provides high-fidelity segmentation suitable for cell nuclei, making it particularly effective in histopathology image analysis.

Although star-convex polygons have been previously investigated for object detection in natural images [27], they were found to be less suitable for generic object classes like people or vehicles. In contrast, in histopathology, Stardist’s ability to segment cell nuclei has encouraged further research, particularly for HER2-SISH image analysis.

Recently, ref. [28] assessed Stardist in segmenting nuclei in CellSearch images, specifically in peripheral blood and DLA samples. Their study highlighted significant improvements in segmenting CellSearch images, resulting in more accurate counts of circulating tumor cells (CTCs). These findings emphasize Stardist’s potential for nuclei localization using star-convex polygons, making it a promising tool for advanced histopathology analysis, including applications in HER2-SISH images.

## 3. Materials and Methods

**Figure 3:**
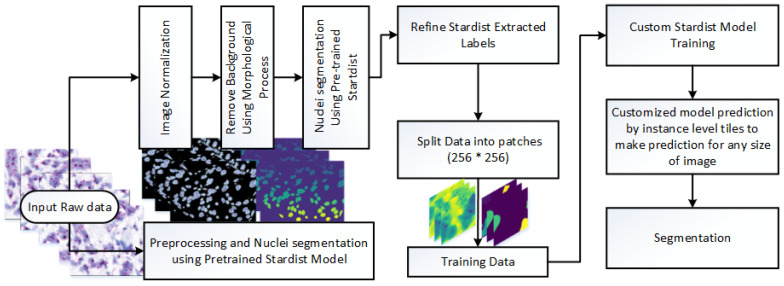
Workflow of our proposed method for SISH nuclei detection. Main steps include: (1) pre-processing, involving sub-steps for rough nuclei segmentation and training data creation, and (2) training the Stardist model for nuclei detection and segmentation.

This study presents a novel framework for nuclei detection in HER2-SISH-stained whole slide images (WSIs) using a two-stage deep learning approach. Our methodology combines a pretrained Stardist model with morphological preprocessing and fine tuning on expert-annotated ROIs. The workflow is illustrated in Figure 3, with each component detailed in the following subsections.

### 3.1. Dataset Description

**Figure 4:**
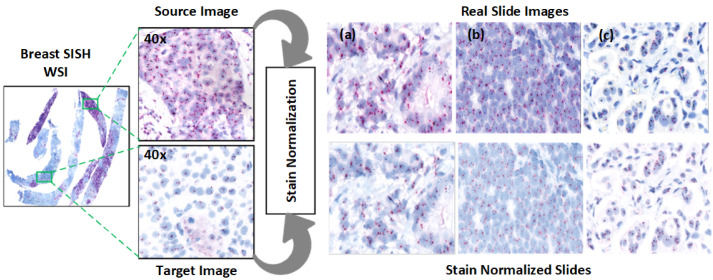
Stain normalization on selected WSI patches. Subfigures (**a**–**c**) show original HER2-SISH image regions, while the corresponding stain-normalized outputs are displayed directly below each original patch.

A total of 20 HER2-SISH whole slide images (WSIs) were obtained from breast cancer biopsy cases at the University of Malaya Medical Center (UMMC), comprising 8 HER2-amplified and 12 non-amplified cases. The biopsy samples were stained using the SISH protocol and scanned using a 3DHistech Pannoramic DESK scanner, producing high-resolution WSIs with dimensions ranging from 80,000×200,000 pixels. From each WSI, expert pathologists selected five regions of interest (ROIs), resulting in 100 ROIs in total. The ROI dimensions varied from 859 × 755 to 5451 × 3136 pixels. Figure 4 illustrates an example ROI following stain normalization, and Table 2 summarizes the dataset characteristics.

The relatively small dataset size (20 WSIs with 100 ROIs) reflects the significant manual effort required for expert annotation and the high-resolution nature of the SISH images. Despite its size, this curated dataset enabled the model to generalize well, as evidenced by strong F1-scores across expert evaluations.

The dataset was split into an 80:20 ratio for training and testing. The training set consisted of 80 ROIs, used to create labels and fine tune a custom Stardist model. Initial annotations were generated using a pretrained Stardist model and refined through manual corrections. The remaining 20 ROIs were reserved for evaluation, both visually and using expert-marked nuclei as ground truth.

Inclusion criteria required archived HER2-SISH WSIs from pathologically confirmed breast cancer cases with complete HER2 scoring records. Exclusion criteria eliminated samples with poor tissue preservation, insufficient metadata, or image artifacts affecting nuclei visibility. ROIs were selected only if they showed clear nuclear morphology, good staining contrast, and minimal overlapping. Regions with ambiguous features or nuclei clustering were excluded.

Three pathologists participated in the ROI selection process, including a senior consultant with extensive experience in breast pathology. To mitigate inter-observer variability, ROIs were initially selected independently by two pathologists and later reviewed and finalized through consensus with the senior pathologist.

### 3.2. Cytomine Platform for ROI Selection and Real-Time Segmentation

To make our framework accessible for clinical application, we implemented it on the Cytomine platform [29]. Cytomine allows pathologists to select ROIs directly from WSIs, enabling them to interactively apply our model for real-time nuclei segmentation (see Figure 2). This integration with Cytomine streamlines the HER2 scoring process by allowing pathologists to focus on specific regions, thus enhancing diagnostic efficiency. Real-time feedback from pathologists also provides a mechanism to refine segmentation accuracy and ensure clinical relevance. ROIs were included only if they exhibited clear nuclear morphology with adequate staining contrast and no overlapping nuclei clusters. Excluded regions included artifacts, ambiguous staining, and overlapping nuclei unsuitable for HER2/CEN17 quantification. To reduce inter-observer variability, an initial set of candidate ROIs was independently selected by two junior pathologists and reviewed by a senior pathologist. Final ROIs were chosen through consensus agreement.

### 3.3. Ground Truth Preparation

To create reliable ground truth labels, we developed a preprocessing method that includes image normalization and nuclei region extraction. First, images were normalized to standardize contrast, and then background removal was applied to enhance nuclei visibility.

#### 3.3.1. Stain Normalization

Normalization addresses the contrast variability in HER2-SISH images. We used the Macenko normalization algorithm [30], which falls under unsupervised normalization techniques:

1. Stain Vector Estimation: Singular value decomposition (SVD) was applied to foreground pixels to estimate the SISH stain vectors.
2. Intensity Correction: Intensity variations, arising from staining and procedural factors, were corrected.
3. Projection to Reference Image: Images were projected to a reference image to maintain consistent color properties across normalized images.

The Macenko algorithm operates by converting RGB vectors to optical density (OD) values using $OD=-\log_{10}I$, allowing a linear combination of stains to yield OD values. An open-source Python 3.11 implementation of the Macenko method was utilized [31].

Normalization effectiveness was confirmed through statistical testing, with results indicating that the Macenko method aligns image contrast with the reference image (Table 3). Visual examples are shown in Figure 4.

#### 3.3.2. Extraction of Image Foreground

**Figure 5:**
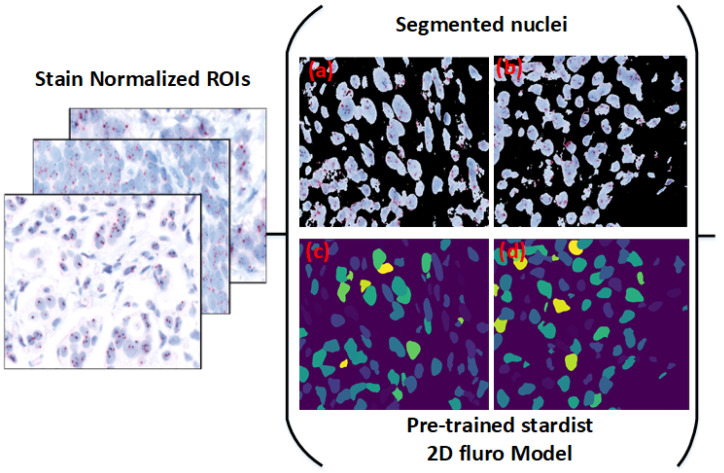
Preprocessed images after background removal and rough nuclei segmentation. Subfigures (**a**,**b**) show example HER2-SISH image patches after background removal using thresholding and morphological operations. Subfigures (**c**,**d**) illustrate the corresponding rough nuclei segmentation results obtained post stain normalization.

Following normalization, background removal was applied to improve nuclei visibility, facilitating accurate segmentation with Stardist. Histogram equalization and grayscale conversion were used to enhance contrast, followed by mask creation using thresholding and morphological operations. Figure 5 shows the effect of these preprocessing steps.

The thresholding method involved both global and local adjustments to distinguish nuclei intensity. Morphological refinements were incorporated to improve boundary accuracy, resulting in clearer nuclei separation, as illustrated in Figure 5.

### 3.4. Stardist Model for Nuclei Segmentation

Nuclei detection and segmentation in HER2-SISH images are crucial for HER2 scoring and analysis. Stardist [25,32] addresses segmentation challenges by predicting nuclei shapes as star-convex polygons, facilitating precise segmentation in 2D and 3D volumes.

**Figure 6:**
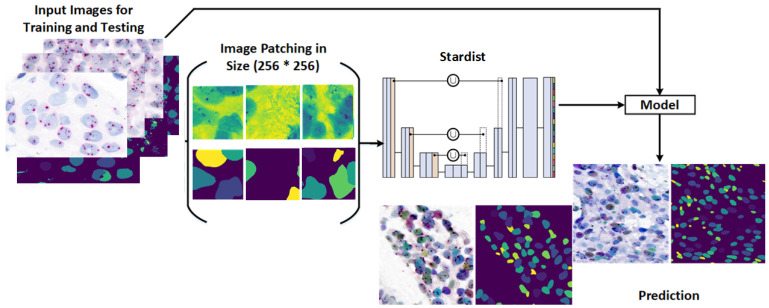
Stepwise workflow for training the custom Stardist model and resulting outcomes.

Originally designed for cell nuclei segmentation, Stardist was adapted here for HER2-SISH nuclei with a fine-tuning process. Figure 6 illustrates the workflow used for training and applying the Stardist model in our study.

Key features of the Stardist model include:

1. CNN Architecture: Built on a U-Net base, Stardist utilizes a contracting path for context capture and an expansive path for localization.
2. StarConvex Shape Prediction: Each image tile is divided into overlapping tiles, predicting object shapes and distance maps that refine boundary accuracy.
3. Fine-Grained Segmentation: Unlike bounding box predictions, Stardist achieves pixel-level boundary precision for accurate object shape capture.
4. Implementation: Our Stardist model was implemented in TensorFlow and fine tuned on the expert-annotated dataset.

### 3.5. Separating Overlapping Nuclei Using Pretrained Stardist

Traditional watershed methods produce excessive segmentation artifacts in clustered regions. To address this, we applied a pretrained Stardist model, specifically the Versatile fluorescent nuclei model, which performed effectively for HER2-SISH nuclei segmentation based on visual and expert evaluations.

A custom wrapper function was used to normalize the input, initialize Stardist, and generate segmentation masks. Figure 5 (bottom right) shows sample nuclei region predictions obtained from this model.

#### Post Processing

**Figure 7:**
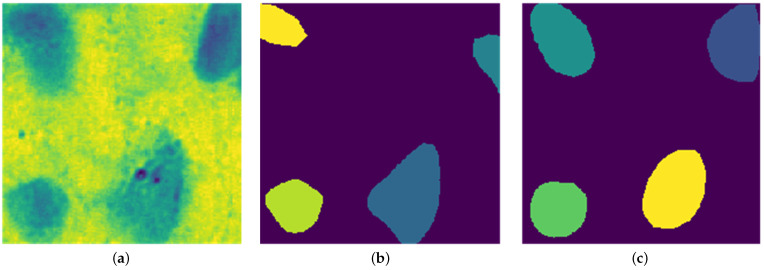
Predicted Stardist image and custom-trained Stardist image: (**a**) 256 × 256 patch of training data, (**b**) curated ground truth label image for model training, (**c**) segmented nuclei image.

Post-processing refines Stardist predictions by removing extraneous small regions and dividing images into 256 × 256 patches, enhancing training efficiency and compatibility with Stardist’s settings. Figure 7 shows the visual comparison between predicted nuclei and ground truth label patches after fine tuning.

## 4. Experimental Setup

This section presents the results obtained by applying our proposed methodology on the HER2-SISH dataset and evaluates its performance against expert-marked, validated regions. As the first computational approach for nuclei detection in HER2-SISH stains, our results focus on object-level detection of nuclei rather than precise boundary segmentation. Consequently, comparisons with existing methods are not applicable. The section is structured into the following subsections.

### 4.1. Network Implementation Details

Our proposed framework was implemented on the Google Colab platform using TensorFlow [33]. The custom Stardist model employed in the two-stage framework was trained with a learning rate of 0.0001, optimized using a stochastic gradient descent optimizer to minimize the loss function. The batch size was set to 4, with a momentum of 0.9. To optimize memory usage and enhance training efficiency, particularly given GPU constraints, the input image size was fixed at 256 × 256. During training, we augmented the dataset by applying random rotations, horizontal flips, and vertical flips. The model was trained for 100 epochs, with a total runtime of approximately 2 h on the Colab environment. While our approach is versatile and not limited to specific regression or classification models, we adopted Stardist, which is built on the U-Net architecture [34], as the basis for our model.

#### 4.1.1. Model Training

To streamline the training process, all data was divided into 256 × 256 patches, aligning with the default input requirements of the Stardist model. The training dataset was further expanded with synthetic augmentations, generating an additional 3650 patches of the same size. This brought the total training set to 6650 patches. Testing did not require preprocessing; ROIs were passed directly to the trained model. An 80:20% train–validation split was applied. To optimize object probability predictions, we utilized binary cross-entropy loss, while mean absolute error loss was used for polygon distances, weighted by ground truth object probabilities. Pixels with zero probability did not contribute to the loss function, and those near the object center were prioritized, enhancing boundary delineation accuracy.

**Figure 8:**
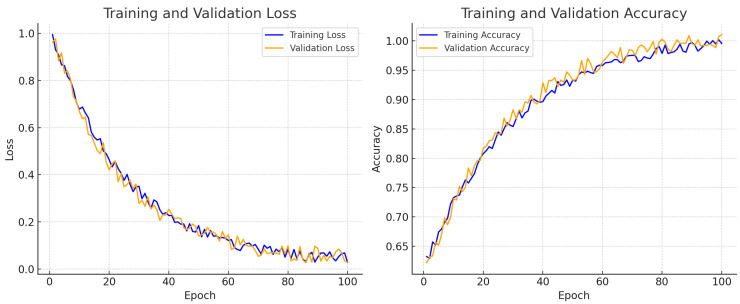
Training and validation curves for loss and accuracy across 100 epochs. The plots simulate typical learning dynamics observed during fine tuning of the Stardist model, reflecting model convergence and generalization performance.

Figure 8 illustrates the simulated training and validation loss and accuracy curves over 100 epochs, reflecting the convergence behavior of the fine-tuned Stardist model.

#### 4.1.2. Testing

The Stardist model’s default test function uses non-maximum suppression (NMS), based on a greedy approach inspired by previous work [35,36,37]. This method retains only polygons with the highest object probabilities within a region, excluding lower-probability predictions. Polygons associated with pixels exceeding a specific object probability threshold were retained, and polygon intersections were resolved using standard clipping methods. Centroids for nuclei detection were identified by overlapping polygons, facilitating robust nuclei detection.

### 4.2. Evaluation Metrics

For comprehensive evaluation of nuclei detection, it is essential to account for both object-level and pixel-level errors. Due to the absence of clearly defined nuclei boundaries in our dataset, we primarily focused on object-level penalties. However, pixel-level penalties are important for maintaining detailed boundaries, as they influence the size and shape of nuclei masks. Following [18], our evaluation criteria for nuclei detection address three potential types of errors:

1. Correct segmentation of overlapping nuclei (True Positive, TP),
2. Segmentation of a single nucleus as multiple regions (False Positive, FP),
3. Missed detection of ground truth nuclei (False Negative, FN).

The results of these evaluations are presented in Table 4 and Table 5, where Table 4 provides quantitative assessments based on visually identified nuclei (ground truth, GT) and Table 5 evaluates model-detected nuclei compared to expert-annotated regions.

For pixel-level evaluations, commonly used metrics such as precision, recall, and F1-score [38] were computed based on the following equations:

(1) $$precision=\frac{TP}{TP+FP}$$

(2) $$recall=\frac{TP}{TP+FN}$$

(3) $$F1_{score}=\frac{2TP}{2TP+FN+FP}$$

**Figure 9:**
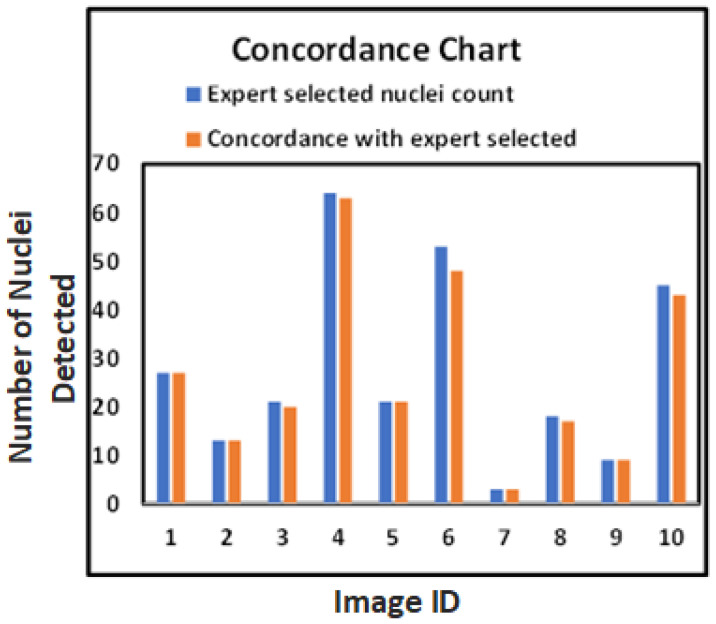
Bar chart comparing the number of nuclei detected by the model (orange bars) against expert-marked nuclei (blue bars) across test images. The X-axis represents Image IDs, and the Y-axis indicates the number of nuclei. The chart demonstrates that our method closely aligns with expert annotations, supporting the effectiveness of the proposed segmentation approach.

These metrics provide a comprehensive assessment of the model’s performance in detecting nuclei. Figure 9 visualizes the model’s performance, demonstrating that our approach closely aligns with expert-marked nuclei regions and visually identified nuclei without classification.

## 5. Results and Discussion

This section presents the results of our proposed methodology for computational analysis and nuclei detection in HER2-SISH images. Given that this approach is novel, there is no direct comparison with existing methods; hence, we compare our results against expert-marked images provided by senior pathologists. A total of 20 images were marked by pathologists, following their established protocols for HER2 scoring from HER2-SISH stains. Our analysis, described in the following subsections, evaluates the results both visually and quantitatively, demonstrating that our proposed method effectively supports HER2-SISH image analysis.

Our framework is already integrated within the Cytomine platform, which is compatible with clinical digital pathology systems. Pathologists can select ROIs interactively, apply real-time segmentation, and incorporate the results into HER2 scoring workflows. This reduces diagnostic time and enhances reproducibility, making it feasible for deployment in clinical settings with digital infrastructure.

### 5.1. Objective Evaluation

Objective evaluation was conducted on 20% of the dataset, primarily due to the limited availability of expert-marked data and the large image sizes, ranging from 859 × 755 to approximately 5451 × 3136 pixels per ROI. Two evaluation approaches were used: one adhering to expert criteria and the other based on visual similarity to nuclei. The expert criteria included only nuclei regions with at least two red signals, non-overlapping, and non-border-touching, as singular nuclei are particularly suitable for HER2 quantification [39]. The second approach considered any object resembling a nucleus, without additional constraints. Table 4 presents visual nuclei detection counts based on visually identified regions, without strict expert constraints.

The evaluation metrics—recall, precision, and F1-score—had mean values of 97.9%, 98.3%, and 98.12%, respectively, for the test images (Table 4). Individual metrics were computed for each test image, showing consistent model performance. For expert-marked, medically significant nuclei, recall, precision, and F1-score reached 98.5%, 96.5%, and 97.42% (Table 5), indicating the model’s reliability for identifying critical nuclei. These results demonstrate a high concordance between model-detected nuclei and expert annotations, suggesting that our method is dependable for clinically relevant nuclei detection.

These results provide a foundation for reliable and reproducible HER2 quantification, as shown in Table 4 and Table 5, and the evaluation graph in Figure 9, which highlights the close alignment of model-detected and expert-marked nuclei regions.

**Figure 10:**
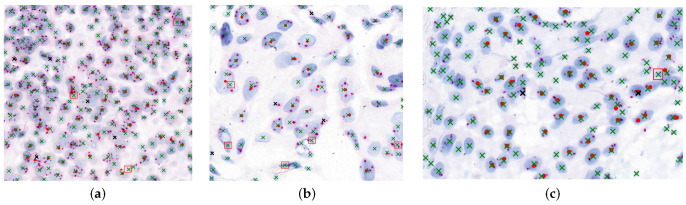
Visualization of nuclei detection results in HER2-SISH image samples. Subfigures (**a**–**c**) present three different HER2-SISH image patches with overlapped and clustered nuclei regions, highlighting detection results. In each image, red dots indicate expert-targeted nuclei used for diagnosis, green “x” marks represent model-detected nuclei, black “x” marks indicate missed nuclei, and red bounding boxes highlight oversegmented regions.

Figure 10 illustrates the agreement between model-detected nuclei and expert-marked regions essential for diagnosis. Red dots represent expert-targeted nuclei, green “x” marks are model-detected nuclei, black “x” marks indicate missed detections, and red boxes denote oversegmented regions.

The bar chart in Figure 9 further confirms the close match between model-detected nuclei and expert annotations. Our method generalizes well across both visual and quantitative evaluations, even with limited annotated data, as demonstrated at the patch level in Figure 7.

### 5.2. Comparison with Baseline Methods

To evaluate the effectiveness of our fine-tuned Stardist model, we compared it against two baseline approaches: (i) a conventional nuclei detection method based on marker-controlled watershed segmentation as proposed in our prior work [40], and (ii) the pretrained Stardist model without fine tuning. Both baseline methods were applied to the same annotated dataset for consistency.

The fine-tuned Stardist model outperformed the other methods in terms of nuclei detection accuracy and F1-score, particularly in challenging regions with clustered or overlapping nuclei. This demonstrates the benefit of domain-specific fine tuning on expert-annotated SISH ROIs. The detailed quantitative results are summarized in Table 6.

## 6. Limitations

While the proposed computational framework for HER2 status assessment demonstrates significant advancements in automating SISH image analysis, there are several limitations. The dataset used in this study consists of a limited number of cases (20 WSIs with 100 ROIs). Although the results show high correlation with manual assessments, a larger and more diverse dataset is necessary to validate the robustness and generalizability of the model across various patient demographics and staining conditions. This study focuses primarily on the HER2/CEP17 ratio as a determinant for HER2 amplification status. However, the ASCO/CAP 2018 guidelines also consider the absolute HER2 copy number. Future work should aim to incorporate this additional criterion for a more comprehensive assessment.

## 7. Conclusions

This study presents a robust and scalable framework for nuclei detection in HER2-SISH whole slide breast tissue images, leveraging expert-marked annotations for both HER2-amplified and non-amplified regions. By integrating a pretrained Stardist model with traditional image processing and fine tuning on a domain-specific dataset, the proposed approach addresses key challenges in SISH-stained nuclei segmentation, including morphological complexity, stain variability, and clustered nuclei.

Our method demonstrated superior performance over both the pretrained Stardist model and a conventional watershed segmentation approach from our previous work [40]. Quantitative results confirm its effectiveness, achieving an average F1-score of 98.1% for visually assessed nuclei and 97.4% for expert-marked nuclei across 20 test images. These improvements were further supported by training–validation accuracy and loss curves, which illustrated stable convergence throughout the training process.

Currently in the testing phase, the framework has been integrated with the Cytomine platform to facilitate clinical deployment. This paves the way for real-time nuclei detection and HER2 scoring support within digital pathology workflows, offering greater consistency and efficiency for pathologists.

Future work will focus on expanding the dataset with more expert-annotated cases, enabling broader benchmarking and generalization. Additionally, we aim to extend the framework to include nuclei classification, signal quantification, and segmentation-based HER2 ratio calculation, contributing toward the automation of HER2 diagnostic scoring protocols.

## Figures and Tables

**Table 1:** Comparison of different ISH stains for HER2 scoring based on their characteristics, advantages, and limitations.

Sr. No.	Characteristics	FISH	CISH	SISH
1	Gold standard for HER2 scoring	**✓**	**✓**	**✓**
2	Low costs and labor	**✗**	**✓**	**✓**
3	Do not need specialized instruments	**✗**	**✓**	**✓**
4	Practical convenience and efficiency	**✗**	**✗**	**✓**
5	Samples do not decay over time	**✗**	**✗**	**✓**
6	Do not require longer time for staining and scoring slides	**✗**	**✓**	**✓**

**Table 2:** Dataset summary for HER2 scoring.

Sr. No.	Dataset Details	No. of Images	Size of Image
1	Total WSI imaes	20	WSI image size are varying in range of 3Giga byte
2	Extracted Patches of WSI	100	From each WSI 5 regions and they varying in size. Rough size range of 714 × 560 to 5451 × 3136

**Table 3:** SSIM-based evaluation of the normalization approach for HER2-SISH stain. SSIM (Structural Similarity Index Measure) quantifies image similarity based on luminance, contrast, and structure. Higher SSIM values indicate that the stain-normalized images closely match the reference in terms of visual fidelity and structural preservation.

Sr. No.	Image ID	SSIM vs. Ref Image SSIM	Normalized SSIM vs. Ref Image SSIM
1	Image (a)	0.67	0.76
2	Image (b)	0.68	0.75
3	Image (c)	0.66	0.73

**Table 4:** Quantitative evaluation based on model-detected nuclei, considering all objects resembling nuclei as ground truth (GT) and computing TP, FP, and FN. Objective evaluations are derived from these counts.

Sr. No.	Image ID	Visual Nuclei	Model Detected			Evaluation Metrices		
			**TP**	**FP**	**FN**	**Recall**	**Precision**	**F1**
1	169284650	146	143	3	3	97.9	97.9	97.9
2	169282562	118	117	1	0	100.0	99.2	99.6
3	169284039	77	67	4	5	93.1	94.4	93.7
4	169285655	207	202	5	3	98.5	97.6	98.1
5	168138203	62	61	0	1	98.4	100.0	99.2
6	168138509	153	148	4	5	96.7	97.4	97.0
7	169262486	43	42	0	1	97.7	100.0	98.8
8	168138726	66	65	1	0	100.0	98.5	99.2
9	169283283	86	84	0	2	97.7	100.0	98.8
10	169284222	126	125	2	1	99.2	98.4	98.8
11	168138775	168	164	4	0	100.0	97.6	98.8
12	169262529	24	21	3	0	100.0	87.5	93.3
13	168138327	59	55	4	0	100.0	93.2	96.5
14	169282117	62	59	2	0	100.0	96.7	98.3
15	169284146	28	31	0	3	91.2	100.0	95.4
16	169285625	203	201	4	3	98.5	98.0	98.3
17	169282871	62	61	1	0	100.0	98.4	99.2
18	169283207	157	156	1	0	100.0	99.4	99.7
19	169283699	167	161	7	2	98.8	95.8	97.3
20	169284611	297	300	0	3	99.0	100.0	99.5
**Mean**						**98.3**	**97.5**	**97.87**
**Std**						**2.4**	**3.0**	**1.9**

**Table 5:** Quantitative evaluation of the model’s ability to detect expert-marked nuclei, with TP, FP, and FN computed based on comparison with expert annotations.

Sr. No.	Image ID	Expert Nuclei	Model Detected			Evaluation Metrices		
			**TP**	**FP**	**FN**	**Recall**	**Precision**	**F1**
1	169284650	27	27	0	0	100.0	100.0	100.0
2	169282562	13	13	0	0	100.0	100.0	100.0
3	169284039	21	20	0	1	95.2	100.0	97.6
4	169285655	64	62	2	2	96.9	96.9	96.9
5	168138203	21	20	0	1	95.2	100.0	97.6
6	168138509	53	49	4	1	98.0	92.5	95.1
7	169262486	3	3	0	0	100.0	100.0	100.0
8	168138726	18	16	2	0	100.0	88.9	94.1
9	169283283	9	8	1	0	100.0	88.9	94.1
10	169284222	44	43	1	0	100.0	97.7	98.9
11	168138775	41	41	0	0	100.0	100.0	100.0
12	169262529	4	4	0	0	100.0	100.0	100.0
13	168138327	14	14	0	0	100.0	100.0	100.0
14	169282117	8	6	2	0	100.0	75.0	85.7
15	169284146	11	11	0	0	100.0	100.0	100.0
16	169285625	66	65	1	0	100.0	98.5	99.2
17	169282871	10	10	0	0	100.0	100.0	100.0
18	169283207	22	22	0	0	100.0	100.0	100.0
19	169283699	8	7	1	0	100.0	87.5	93.3
20	169284611	40	40	0	2	95.2	100.0	97.6
**Mean**						**99.0**	**96.3**	**97.50**
**Std**						**1.8**	**6.6**	**3.6**

**Table 6:** Quantitative comparison of nuclei detection methods on HER2-SISH images.

Method	Precision (%)	Recall (%)	F1-Score (%)
Watershed (Rehman et al., 2023) [40]	90.2	88.7	89.4
Pre-trained Stardist	94.1	95.8	94.9
Fine-tuned Stardist (Proposed)	**97.5**	**99.0**	**98.2**

## Data Availability

The dataset used in this study consists of anonymized HER2-SISH whole slide images (WSIs) collected from the University of Malaya Medical Center (UMMC), including 100 expert-annotated regions of interest (ROIs) from 20 WSIs. Due to institutional privacy policies and ethical considerations, the dataset is not publicly available. However, it may be made available by the authors upon reasonable request and with explicit permission from UMMC, depending on the intended use.

## Abbreviations

Commonly used acronyms throughout the manuscript:

HER2	Human Epidermal Growth Factor Receptor 2
SISH	Silver In Situ Hybridization
WSI	Whole Slide Image
ROI	Region of Interest
IHC	Immunohistochemistry
CNN	Convolutional Neural Network
GT	Ground Truth
OD	Optical Density
TP	True Positive
FP	False Positive
FN	False Negative
F1	F1-Score (Harmonic mean of precision and recall)

## References

[1] Siegel R., Miller K., Jemal A. (2020). Cancer statistics, 2020. CA Cancer J. Clin. Am. Cancer Soc..

[2] Fitzmaurice C., Akinyemiju T.F., Al Lami F.H., Alam T., Alizadeh-Navaei R., Allen C., Alsharif U., Alvis-Guzman N., Amini E., Anderson B.O. (2018). Global, regional, and national cancer incidence, mortality, years of life lost, years lived with disability, and disability-adjusted life-years for 29 cancer groups, 1990 to 2016: A systematic analysis for the global burden of disease study. JAMA Oncol..

[3] Kesson E.M., Allardice G.M., George W.D., Burns H.J., Morrison D.S. (2012). Effects of multidisciplinary team working on breast cancer survival: Retrospective, comparative, interventional cohort study of 13 722 women. BMJ.

[4] Veta M., Pluim J.P., Van Diest P.J., Viergever M.A. (2014). Breast cancer histopathology image analysis: A review. IEEE Trans. Biomed. Eng..

[5] Oliveira S.P., Ribeiro Pinto J., Gonçalves T., Canas-Marques R., Cardoso M.J., Oliveira H.P., Cardoso J.S. (2020). Weakly-supervised classification of HER2 expression in breast cancer haematoxylin and eosin stained slides. Appl. Sci..

[6] Rakha E.A., Tan P.H., Quinn C., Provenzano E., Shaaban A.M., Deb R., Callagy G., Starczynski J., Lee A.H., Ellis I.O. (2022). UK recommendations for HER2 assessment in breast cancer: An update. J. Clin. Pathol..

[7] Goddard K., Weinmann S., Richert-Boe K., Chen C., Bulkley J., Wax C. (2012). HER2 evaluation and its impact on breast cancer treatment decisions. Public Health Genom..

[8] Ahn S., Woo J.W., Lee K., Park S.Y. (2020). HER2 status in breast cancer: Changes in guidelines and complicating factors for interpretation. J. Pathol. Transl. Med..

[9] Williams S., Henricks W.H., Becich M.J., Toscano M., Carter A.B. (2010). Telepathology for patient care: What am I getting myself into?. Adv. Anat. Pathol..

[10] Sucaet Y., Waelput W. (2014). Digital Pathology.

[11] Stoler M.H., Ronnett B.M., Joste N.E., Hunt W.C., Cuzick J., Wheeler C.M. (2015). The interpretive variability of cervical biopsies and its relationship to HPV status. Am. J. Surg. Pathol..

[12] Pantanowitz L., Szymas J., Yagi Y., Wilbur D. (2012). Whole slide imaging for educational purposes. J. Pathol. Inform..

[13] Papouchado B.G., Myles J., Lloyd R.V., Stoler M., Oliveira A.M., Downs-Kelly E., Morey A., Bilous M., Nagle R., Prescott N. (2010). Silver in situ hybridization (SISH) for determination of HER2 gene status in breast carcinoma: Comparison with FISH and assessment of interobserver reproducibility. Am. J. Surg. Pathol..

[14] Gurcan M.N., Boucheron L.E., Can A., Madabhushi A., Rajpoot N.M., Yener B. (2009). Histopathological image analysis: A review. IEEE Rev. Biomed. Eng..

[15] Xing F., Yang L. (2016). Robust nucleus/cell detection and segmentation in digital pathology and microscopy images: A comprehensive review. IEEE Rev. Biomed. Eng..

[16] Xing F., Xie Y., Yang L. (2015). An automatic learning-based framework for robust nucleus segmentation. IEEE Trans. Med. Imaging.

[17] Yang X., Li H., Zhou X. (2006). Nuclei segmentation using marker-controlled watershed, tracking using mean-shift, and Kalman filter in time-lapse microscopy. IEEE Trans. Circuits Syst. I Regul. Pap..

[18] Kumar N., Verma R., Sharma S., Bhargava S., Vahadane A., Sethi A. (2017). A dataset and a technique for generalized nuclear segmentation for computational pathology. IEEE Trans. Med. Imaging.

[19] Cui Y., Zhang G., Liu Z., Xiong Z., Hu J. (2019). A deep learning algorithm for one-step contour aware nuclei segmentation of histopathology images. Med. Biol. Eng. Comput..

[20] Naylor P., Laé M., Reyal F., Walter T. (2018). Segmentation of nuclei in histopathology images by deep regression of the distance map. IEEE Trans. Med. Imaging.

[21] Khoshdeli M., Parvin B. (2018). Deep learning models delineate multiple nuclear phenotypes in H&E stained histology sections. arXiv.

[22] Mahmood F., Borders D., Chen R.J., McKay G.N., Salimian K.J., Baras A., Durr N.J. (2019). Deep adversarial training for multi-organ nuclei segmentation in histopathology images. IEEE Trans. Med. Imaging.

[23] Xu J.S., Lei L., Shuxi Z., Yameng Z., Guohua S., Yucheng L., Jie G., Yu F. (2025). PointFormer: Keypoint-Guided Transformer for Simultaneous Nuclei Segmentation and Classification in Multi-Tissue Histology Images. IEEE Trans. Med. Imaging.

[24] Rauf Z., Khan A.R., Khan A. (2024). Channel Boosted CNN-Transformer-based Multi-Level and Multi-Scale Nuclei Segmentation. arXiv.

[25] Weigert M., Schmidt U., Haase R., Sugawara K., Myers G. Star-convex polyhedra for 3D object detection and segmentation in microscopy. Proceedings of the IEEE/CVF Winter Conference on Applications of Computer Vision.

[26] Schmidt U., Weigert M., Broaddus C., Myers G. (2018). Cell detection with Star-convex polygons. arXiv.

[27] Jetley S., Sapienza M., Golodetz S., Torr P.H. Straight to shapes: Real-time detection of encoded shapes. Proceedings of the IEEE Conference on Computer Vision and Pattern Recognition.

[28] Stevens M., Nanou A., Terstappen L.W., Driemel C., Stoecklein N.H., Coumans F.A. (2022). StarDist Image Segmentation Improves Circulating Tumor Cell Detection. Cancers.

[29] Rubens U., Hoyoux R., Vanosmael L., Ouras M., Tasset M., Hamilton C., Longuespée R., Marée R. (2019). Cytomine: Toward an open and collaborative software platform for digital pathology bridged to molecular investigations. PROTEOM—Clin. Appl..

[30] Titford M., Bowman B. (2012). What may the future hold for histotechnologists?. Lab. Med..

[31] Khan A.M., Rajpoot N., Treanor D., Magee D. (2014). A Nonlinear Mapping Approach to Stain Normalization in Digital Histopathology Images Using Image-Specific Color Deconvolution. IEEE Trans. Biomed. Eng..

[32] Zhao Y., Fu C., Zhang W., Ye C., Wang Z., Ma H.-f. (2022). Automatic Segmentation of Cervical Cells Based on Star-Convex Polygons in Pap Smear Images. Bioengineering.

[33] Abadi M., Barham P., Chen J., Chen Z., Davis A., Dean J., Devin M., Ghemawat S., Irving G., Isard M. TensorFlow: A System for Large-Scale Machine Learning. Proceedings of the 12th USENIX Symposium on Operating Systems Design and Implementation (OSDI 16).

[34] Ronneberger O., Fischer P., Brox T. U-net: Convolutional networks for biomedical image segmentation. Proceedings of the Medical Image Computing and Computer-Assisted Intervention–MICCAI 2015: 18th International Conference.

[35] Liu W., Anguelov D., Erhan D., Szegedy C., Reed S., Fu C.Y., Berg A.C. Ssd: Single shot multibox detector. Proceedings of the Computer Vision–ECCV 2016: 14th European Conference.

[36] Redmon J., Divvala S., Girshick R., Farhadi A. You only look once: Unified, real-time object detection. Proceedings of the IEEE Conference on Computer Vision and Pattern Recognition.

[37] Ren S., He K., Girshick R., Sun J. (2015). Faster r-cnn: Towards real-time object detection with region proposal networks. Adv. Neural Inf. Process. Syst..

[38] Sasaki Y. (2007). The truth of the F-measure. Teach Tutor Mater..

[39] Hossain M.S., Syeed M.M., Fatema K., Hossain M.S., Uddin M.F. (2022). Singular nuclei segmentation for automatic HER2 quantification using CISH whole slide images. Sensors.

[40] Rehman Z.U., Ahmad Fauzi M.F., Wan Ahmad W.S.H.M., Cheah P.L., Looi L.M., Toh Y.F., Abas F.S. Nuclei Detection in HER2-SISH Histopathology Images. Proceedings of the 2023 IEEE 2nd National Biomedical Engineering Conference (NBEC).

